# Thermal Conductivities of Uniform and Random Sulfur Crosslinking in Polybutadiene by Molecular Dynamic Simulation

**DOI:** 10.3390/polym15092058

**Published:** 2023-04-26

**Authors:** Tannaz Alamfard, Tommy Lorenz, Cornelia Breitkopf

**Affiliations:** Chair of Thermodynamics, Technical University Dresden, 01069 Dresden, Germany; tommy.lorenz@tu-dresden.de (T.L.); cornelia.breitkopf@tu-dresden.de (C.B.)

**Keywords:** equilibrium molecular dynamics simulation (EMD), force field, degree of crosslinking, polybutadiene, thermal conductivity, autocorrelation function, Green–Kubo method

## Abstract

Thermal conductivities of polybutadiene crosslinked with sulfur as a function of the heat flux autocorrelation function by using an equilibrium molecular dynamic (EMD) simulation were investigated. The Green–Kubo method was used to calculate thermal conductivities. All simulations were performed by applying the LAMMPS software (version 3 Mar 2020) package. The united-atom force field (OPLS-UA) from the Moltemplate software (version 2.20.3) was applied in the simulations. The influence of uniform and random distributions of sulfur in polybutadiene on the final value of thermal conductivities was studied by polymeric model structures with similar and variable degrees of crosslinking. The results showed that for identical degrees of crosslinking, the distribution of crosslinkers in the polymeric model structures significantly influenced the final value of thermal conductivity. Moreover, the influence of the crosslinking degree on the final value of thermal conductivity was studied by considering polymeric model structures with different degrees of crosslinking. The results demonstrate that by having a random distribution of sulfur, the thermal conductivity will be enhanced. However, by increasing the degree of crosslinking to the higher percentage in random crosslinked model structures, the value of thermal conductivity drops significantly due to possible higher crystallization of the model structures, which decrease the degree of freedom for phonon contributions.

## 1. Introduction

In recent decades, computational modeling has become common in investigating molecular models representing polymeric structures and rubbers at an atomic scale. Progress in simulation methods and advancements in computational power has enabled scientists to probe polymeric structures at the microscale level with the aim of predicting physical properties of interest. Therefore, some methods including equilibrium molecular dynamics (EMD), non-equilibrium molecular dynamic (NEMD), and Brownian dynamics are established to calculate thermo-mechanical properties of the polymer structures according to their preparation methods. One option to enhance material properties is vulcanization. 

The vulcanization process is induced through the formation of crosslinking bridges between macromolecules. The type of crosslinking and degree of crosslinking determine the thermo-mechanical properties of a system. One of the major vulcanization methods that is frequently used is the vulcanization with sulfur and accelerators [1].

Since that time, there has also been great interest in investigating such structures on a molecular level.

The first attempt to study crosslinking polymeric structures postulated that crosslinking occurs instantaneously, which was reported by Doherty et al. [2]. In addition, Komarov et al. calculated the thermal properties of epoxy resin by applying non-equilibrium molecular dynamic simulations [3]. Moreover, Varshney et al. conducted a non-equilibrium MD simulation to investigate the thermal conductivity of epoxy resins [4]. Selecting a correct force field plays a significant role in the success of MD simulations [5,6,7,8]. The all-atom (AA) model is an appropriate force field to apply to structures because of its capability to take into account more potential interactions [8]. However, MD simulations using an AA model are not suitable for structures that contain a large number of atoms because they need a long simulation time to be run. Therefore, a united-atom model is a more suitable model that increases computational efficiency by decreasing the molecular degrees of freedom [6,9].

In chemical structures that contain hydrogen atoms, in many cases, there is no need to clearly treat hydrogen atoms explicitly, which is mainly because hydrogen atoms move at a high-frequency rate. Thus, the related phenomena confine the study to generate smaller time steps in simulations, which in turn results in restricting the computational speed. When increasing the efficiency of simulations, a united-atom model is a solution to not considering hydrogen atoms explicitly so that they can be considered in combination with the group of heavier atoms that they have attached to [10,11,12,13,14,15,16,17]. United-atom models can be beneficial in enhancing computational efficiency; however, some adjustments must be applied to the united atom to adapt to the system status and the thermodynamics that may be caused by hydrogen bonding [14]. By considering united atoms, the atomistic motions of the structure may be sped up. In addition, the lack of hydrogen may cause a deviation from the real results, which is mainly because of variation in partial charge. There are many accessible force-field options related to polymers, so the first step is to find a suitable force field. Moreover, the potential energy of the structure is related to the atomic coordination of the structure [18]. Most of the MD simulations that have been used in the past to obtain thermal properties of model structures were restricted to simple materials such as metals, pure polymers, and ionic salts [19,20,21]. However, the thermal properties of crosslinked polymeric structures have been investigated recently [22]. The thermal conductivity can be obtained by applying either equilibrium molecular dynamic (EMD) (using the Green–Kubo equation [23]) or using non-equilibrium molecular dynamic (NEMD) simulations [24]. In NEMD simulations, the heat flux is calculated by considering a long polymeric slab with a temperature difference between two ends of the slab, from which the heat difference can be obtained. Varshney et al. [22] made a comparison between NEMD and EMD methods (by applying the Green–Kubo equation) previously. They used these two methods for the same structure and obtained the same result for thermal conductivity.

In this study, the thermal conductivities of polybutadienes were crosslinked with sulfur as a function of heat flux autocorrelation function by using the equilibrium molecular dynamic (EMD) simulation. The Green–Kubo method was used to calculate thermal conductivities. The united-atom force field (OPLS-UA) from the Moltemplate software (version 2.20.3) was applied in all simulations. The main focus was to find structure–property relations, e.g., to describe the dependence of the heat conductivity on structure modifications with respect to uniform or random types of crosslinking, as well as variable degrees of crosslinking. As experimental details for crosslinked polymers are rare, this inverse approach may help to characterize polymer samples by a theoretical approach.

## 2. Materials and Methods

### 2.1. Green–Kubo Method for Calculating Thermal Conductivity

In this article, the thermal conductivity of polybutadiene crosslinked with sulfur was investigated by using an equilibrium molecular dynamic (EMD) method. One approach, which is used here to extract the heat conductivity, is the Green–Kubo method, in which the heat flux along each direction correlates to the thermal conductivity (λ) based on the fluctuation–dissipation principle [23,25]. The thermal conductivity along *x* direction is as follows,

(1) $$K=\frac{V}{k_{B}T^{2}}\int_{0}^{\infty }❬J_{x}\left(t\right)J_{x}\left(0\right)❭dt\;$$

where $J_{x}$, $❬J_{x}\left(t\right)J_{x}\left(0\right)❭$, *V*, *T* and $k_{B}$ are the heat flux along *x* direction, the heat flux autocorrelation function (HFACF), the volume of the structure, the temperature of the structure, and the Boltzmann constant, respectively. Generally, in order to attain the thermal conductivity of an isotropic structure, the average of thermal conductivities along all three directions (*x*, *y*, and *z*) has to be calculated. Therefore, in the current study, a factor of three in the denominator of the Green–Kubo formula is applied to calculate the averages of thermal conductivities along the *x*, *y* and *z* directions.

(2) $$K=\frac{V}{3\;k_{B}T^{2}}\int_{0}^{t_{c}}❬J\left(t\right)J\left(0\right){❭}_{t_{s}}dt\;$$

in the above equation, *J* denotes the heat flux vector, $t_{s}$ is the time interval in which the average of the ensemble for calculating the HFACF is accumulated, and $t_{c}$ is the finite correlation time in which the integration is accomplished. In order to obtain the heat flux vector, there are two approaches possible [26]. The following equation is the most common way of defining the heat flux vector:

(3) $$J=\frac{1}{V}\frac{d}{dt}\overset{N}{\underset{i=1}{\sum }}r^{i}e^{i}\;$$

in the above expression, $r^{i}$, $e^{i}$, and *N* are the position vector of atom *i*, the total energy of atom *i*, and the number of all atoms in the structure, respectively. The energy of each atom can be obtained by adding the amount of kinetic energy and potential energy of the related atom,

(4) $$e^{i}=\frac{1}{2}\;m^{i}\;{\left|\upsilon^{i}\right|}^{2}+U^{i}\;$$

where $e^{i}$, $m^{i}$, $\upsilon^{i}$, and $U^{i}$ denote total energy, mass, velocity, and potential energy of atom *i*.

The total potential energy of atom *i* $\left(U^{i}\right)$ would be exclusively defined based on the interaction potential energy, which will be used in the simulations. These interaction potential energies are composed of bonded and non-bonded interactions. The total potential energy is calculated by adding the following interactions: pair interactions $\left(U_{\mathrm{pair}}\right)$ related to van der Waals potential energy; Coulomb interactions $\left(U_{\mathrm{coulomb}}\right)$; bond interactions $\left(U_{\mathrm{bond}}\right)$ related to covalent bonds; angle interactions $\left(U_{\mathrm{angle}}\right)$; dihedral interaction $\left(U_{\mathrm{dihedral}}\right)$; and improper interactions $\left(U_{\mathrm{improper}}\right)$, as follows,

(5) $$U=U_{\mathrm{pair}}+U_{\mathrm{coulomb}}+U_{\mathrm{bond}}+U_{\mathrm{angle}}+U_{\mathrm{dihedral}}+U_{\mathrm{improper}}.$$

where in each interaction term, there is a specific number of atoms, for instance, three atoms are participating in angle interactions. Therefore, 1/3 of the angle energy is devoted to each of the three atoms in the angle term (the allocation of this energy contribution). Thus, the total potential energy of atom *i* will be obtained by averaging the energy contributions of each atom in the related interaction term.

(6) $$\begin{array}{l} U^{i}=\left[\frac{1}{2}\;\overset{N_{p}}{\underset{n=1}{\sum }}U_{\mathrm{pair}}\;\left(r^{i}\;,\;r^{2}\right)+\frac{1}{2}\;\overset{N_{c}}{\underset{n=1}{\sum }}U_{\mathrm{coulomb}}\;\left(r^{i}\;,\;r^{2}\right)+\frac{1}{2}\;\overset{N_{b}}{\underset{n=1}{\sum }}U_{\mathrm{bond}}\;\left(r^{i}\;,\;r^{2}\right)\;\right]\\ \;\;\;\;\;\;\;\;\;\;\;\;\;\;\;\;\;\;\;\;\;\;\;\;\;\;\;\;\;\;\;\;+[\frac{1}{3}\;\overset{N_{a}}{\underset{n=1}{\sum }}U_{\mathrm{angle}}\;\left(r^{i}\;,\;r^{2}\;,\;r^{3}\right)+\frac{1}{4}\;\overset{N_{d}}{\underset{n=1}{\sum }}U_{\mathrm{dihedral}}\;\left(r^{i}\;,\;r^{2}\;,\;r^{3}\;,\;r^{4}\right)\\ \;\;\;\;\;\;\;\;\;\;\;\;\;\;\;\;\;\;\;\;\;\;\;\;\;\;\;\;\;\;\;\;+\frac{1}{4}\;\overset{N_{i}}{\underset{n=1}{\sum }}U{\;}_{\mathrm{improper}}\;\left(r^{i}\;,\;r^{2}\;,\;r^{3}\;,\;r^{4}\right)] \end{array}$$

where the first set of interactions is related to the van der Waals energy, and the parameters of $N_{p}$, $r^{i}$ and $r^{2}$, respectively, indicate neighbors of atom *i*, the position of atom *i*, and the position of neighbor atoms. The third term is related to the bond contribution of atom *i* with $N_{b}$ bonds. For other terms related to angle, dihedral, and improper interaction, the same parameters exist, including $N_{a}$, $N_{d}$ and $N_{i}$, which are related to atoms that atom *i* is a part of.

The microscopic heat flux vector *J* would be calculated by inserting Equation (4) into Equation (3) and conducting differentiation according to the time,

(7) $$J=\frac{1}{V}\overset{N}{\underset{i=1}{\sum }}\upsilon^{i}e^{i}+\overset{N}{\underset{i=1}{\sum }}S^{i}.\upsilon^{i}$$

In Equation (8), $S^{i}$ indicates the stress tensor for each atom. In order to obtain six components of the symmetric tensor, the parameters *a* and *b* take the values *x*, *y*, *z*.

(8) $$\begin{array}{l} VS_{ab}^{i}=\left[\frac{1}{2}\overset{N_{p}}{\underset{n=1}{\sum }}\left(r_{a}^{i}F_{b}^{i}+r_{a}^{2}F_{b}^{2}\right)+\frac{1}{2}\overset{N_{c}}{\underset{n=1}{\sum }}\left(r_{a}^{i}F_{b}^{i}+r_{a}^{2}F_{b}^{2}\right)+\frac{1}{2}\overset{N_{b}}{\underset{n=1}{\sum }}\left(r_{a}^{i}F_{b}^{i}+r_{a}^{2}F_{b}^{2}\right)\right]\\ \;\;\;\;\;\;\;\;\;\;\;\;\;\;\;\;\;\;\;\;\;\;\;\;\;\;\;\;\;\;\;\;\;\;+[\frac{1}{3}\overset{N_{a}}{\underset{n=1}{\sum }}\left(r_{a}^{i}F_{b}^{i}+r_{a}^{2}F_{b}^{2}+r_{a}^{3}F_{b}^{3}\right)\\ \;\;\;\;\;\;\;\;\;\;\;\;\;\;\;\;\;\;\;\;\;\;\;\;\;\;\;\;\;\;\;\;\;\;+\frac{1}{4}\overset{N_{d}}{\underset{n=1}{\sum }}\left(r_{a}^{i}F_{b}^{i}+r_{a}^{2}F_{b}^{2}+r_{a}^{3}F_{b}^{3}+r_{a}^{4}F_{b}^{4}\right)]\\ \;\;\;\;\;\;\;\;\;\;\;\;\;\;\;\;\;\;\;\;\;\;\;\;\;\;\;\;\;\;\;\;\;\;+\left[\frac{1}{4}\overset{N_{i}}{\underset{n=1}{\sum }}\left(r_{a}^{i}F_{b}^{i}+r_{a}^{2}F_{b}^{2}+r_{a}^{3}F_{b}^{3}+r_{a}^{4}F_{b}^{4}\right)\right] \end{array}$$

The first bracket is composed of three terms, where $F^{i}$ and $F^{2}$ are the forces on the two atoms based on the interaction between them. In addition, there are the same force parameters for the angle, dihedral, and improper interactions of atom *i* that the next interaction sets. In addition, the stress tensor just includes the virial terms, and it does not contain the kinetic energy [27].

### 2.2. Degree of Crosslinking

In this work, the influence of the degree of crosslinking on thermal conductivity is investigated. The degree of crosslinking (DC) can be understood as the number of crosslinked bonds that have been created in the polymeric model structure between the primary polymeric chains via curing agents. The degree of crosslinking is the ratio of the crosslinked monomer numbers to the total number of monomers, as follows,

(9) $$\mathrm{DC}=2N_{\mathrm{CL}}/N_{\mathrm{mono}}$$

where $N_{\mathrm{CL}}$ and $N_{\mathrm{mono}}$ indicate, respectively, the number of crosslinks and the number of all monomers. A factor of two in the numerator shows that each crosslink bond is created by two monomers in two different chains [28].

## 3. Results and Discussion

### 3.1. Polymeric Model Structures for MD Simulations

**Figure 1:**
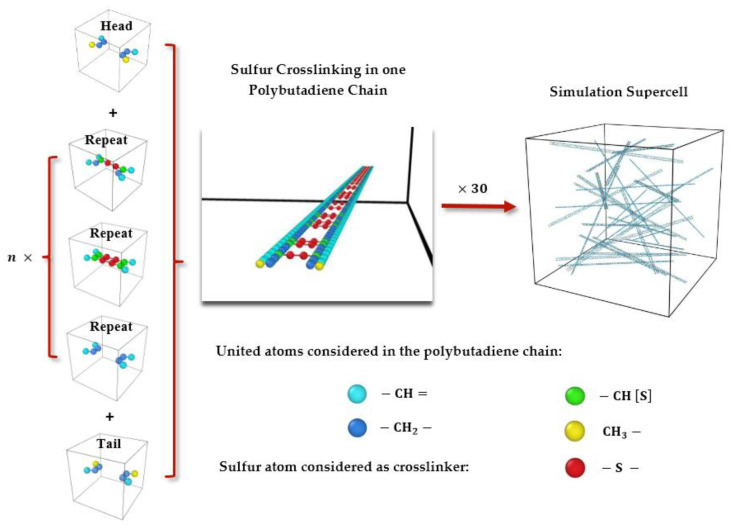
Model process of sulfur crosslinking in polybutadiene.

In this study, a polymeric structure composed of cis-1,4-polybutadiene is considered, which has been crosslinked with sulfur. The molecular model of the related crosslinked polymeric chain was produced by Moltemplate software (version 2.20.3) [29]. In the first step, as illustrated in Figure 1, molecular models of the tail group, the repeat group (body of the chain), and the head group of each chain were generated. To simulate different crosslinking degrees and distributions of sulfur in the final model structures, which will be investigated, three different repeat groups were considered as shown in Figure 1. Then, a head group, a tail group, and a large number of repeat groups were connected to create a crosslinked polybutadiene containing sulfur—either regular or irregular—in a long chain. Each single chain had a specific polymerization degree represented by a composition of 1000 united atoms (containing carbon), as well as a distinct number of sulfur atoms. The number of sulfur atoms in one chain was based on the intended degree of crosslinking that was assumed. Finally, a total of 30 crosslinked single chains were randomly distributed in a box by using Packmol software (version 20.3.5) [30] as displayed in Figure 1. The size of this periodic cubic box must be assumed larger than the size of one chain and large enough to prevent those copies of chains interacting with each other when using periodic conditions. The tolerance factor in Packmol was assumed to be 2.0 Å.

All simulations were performed by applying the LAMMPS software package (version 3 Mar 2020) [31]. A united-atom force field was applied in all simulations. CH–, CH_2_– and CH_3_– groups were treated as “one atom” to not explicitly represent hydrogen atoms with high-frequency motions in the structure and thus to increase computational efficiency. The cutoff distance was set to 10 Å for all simulations. Non-bonded interactions were modeled only via van der Waals interactions using the Lennard–Jones potential. To be able to simulate all possible interactions, a mixed geometric equation was used to describe missing parameters for the Lennard–Jones potential, as not all “pair_styles” commands support the mixing command in LAMMPS, and some mix options are not attainable for specific pair_styles [32]. The parameters of the Lennard–Jones potential and other force fields applied in the MD simulations are summarized in Table 1.

Bond stretching, van der Waals, dihedral and angle interactions were considered with parameters of the united-atom force field (OPLS-UA) obtained by the Moltemplate software (version 2.20.3). In this investigation, a part of the dihedral and angle interactions was not considered because parameters of the united-atom force field do not exist for these combinations. For instance, as shown in Table 1, the force field parameters related to the angle interaction of the combination of united atoms as CH_2_, CH_2_, and CH do not exist (see Vasilev et al. [33]). It showed that this does not have any influence on the final results. Moreover, coefficients for the “special_bonds” command in LAMMPS [32] were set to zero in all simulations.

**Figure 2:**
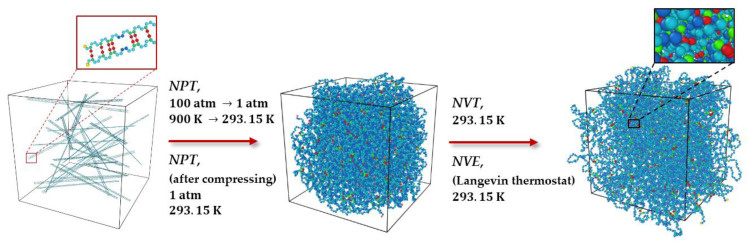
Molecular model of sulfur crosslinking in polybutadiene after equilibrating by applying *NPT*, *NVT*, and *NVE* ensembles.

In order to finally extract the heat conductivity, the normalized heat flux autocorrelation function has to be calculated. In the MD simulations, this can be achieved by applying subsequently different ensembles such as *NPT*, *NVT*, and *NVE* to the periodic box containing the polymeric crosslinked model structures. Thus, the simulation process for the MD runs comprises several steps as shown in Figure 2, which was proceeded for all structures in the same way as described.

After the distribution of the crosslinked chains in the periodic supercell as given in Figure 1, an equilibration process is required to optimize the polymeric model system. By applying the *NPT* ensemble, the polymeric model structure was slowly cooled from high temperatures starting at 900 K and high pressures of 100 atm to the normal temperature of 293.15 K and normal pressure of 1 atm. This process was performed for 200 ps with a time step of 0.2 fs and was performed once.

In the next step, in order to obtain a realistic density, e.g., an equilibrated density of the polymeric model structure at a specific temperature and pressure, Nose and Hoover’s barostat and thermostat [34,35] were applied with damping parameters of 1000 and 100 time steps, respectively. The polymeric model system was therefore simulated for 400 ps with a time step of 0.2 fs in an *NPT* ensemble at a normal temperature of 293.15 K and normal pressure of 1 atm. For each polymeric model structure, different cycles of the *NPT* ensemble were applied to reach an equilibration with respect to the density and temperature.

After equilibration of the density, the energy of the polymeric model structure needs to be equilibrated. Thus, the system was modeled in an *NVT* ensemble at a temperature of ***T*** = 293.15 K for 800 ps with a time step of 0.2 fs. The *NVT* ensemble was applied once to all model structures. After completing the above procedure, a realistic molecular model structure of crosslinked polybutadiene was generated. Starting with these equilibrated model structures, the heat flux autocorrelation function was calculated to finally extract the thermal conductivity.

To calculate this heat flux autocorrelation function, an *NVE* ensemble was applied by using the Langevin thermostat [36]. Then, at each time step, three components of the heat flux in each direction were calculated. The thermal conductivity was derived by applying the Green–Kubo equation for each correlation time interval [23].

The influence of the degree and type of crosslinking on the final thermal conductivities of such model polymeric structures was studied using five different polymer model chains with variable degrees of crosslinking, e.g., representing uniform and non-uniform crosslinked model systems. 

### 3.2. Determination of Thermal Conductivities for Uniform and Non-Uniform Crosslinked Polymers with Varying Degrees of Sulfur Crosslinking Bridges

The influence of the crosslinking type (uniform or non-uniform) as well as the degree of crosslinking (10%, 20%, 30%, 70%) was investigated by generating several polymer model systems. The two values of crosslinking (30% and 70%) may be unrealistic; however, one advantage of modeling structures is the ability to go beyond experimental options and explore structure–property relations. All model systems were simulated according to the MD procedure described in Section 3.1. 

**Figure 3:**
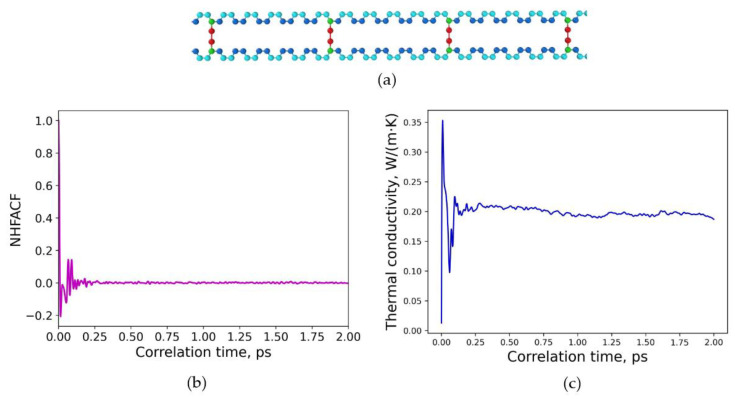
Uniform distribution of sulfur crosslinking in polybutadiene: (**a**) molecular structure of one crosslinked chain, degree of crosslinking is 20%; (**b**) normalized heat flux autocorrelation function (NHFACF) as a function of correlation time with a correlation length of 2.0 ps; (**c**) thermal conductivity as a function of correlation time.

**Figure 4:**
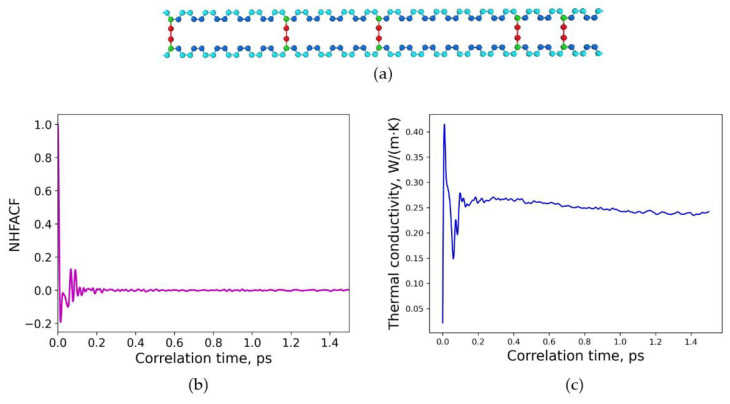
Random distribution of sulfur crosslinking in polybutadiene: (**a**) molecular structure of one crosslinked chain, degree of crosslinking is 20%; (**b**) normalized heat flux autocorrelation function (NHFACF) as a function of correlation time with a correlation length of 1.5 ps; (**c**) thermal conductivity as a function of correlation time.

First, the crosslinking type at a constant degree of crosslinking was within the focus of interest. Therefore, different polybutadiene model structures crosslinked with sulfur were considered, as illustrated in Figure 3a and Figure 4a. The degree of crosslinking was chosen to be constant at 20% for both of these structures. To simulate uniform and non-uniform, e.g., random, structures, the crosslinking sulfur bridges were distributed either uniformly or randomly in the model structures by combining different types of head, repeat, and tail groups according to Figure 1. As experimentalists cannot provide structural information about the detailed incorporation of sulfur into the chains, it is intended to use an inverse procedure here. Therefore, the thermal conductivities of several different model systems are calculated by MD simulations (Section 3.1). The heat flux autocorrelation function is analyzed to give thermal conductivities, and afterwards those values are compared with measured data of polymers with similar sulfur content as Ref. [37]. Thus, a kind of calibration reference may be achieved by this inverse approach to finally characterize real samples.

The thermal conductivities for all polymeric model systems can be derived after the analysis of equilibrated supercells containing a particular uniform or random structure. Therefore, data obtained from the last correlation time interval have been used for calculating the final values of the thermal conductivity as a function of the correlation time.

Figure 3a illustrates a uniform distribution of sulfur crosslinking in polybutadiene with a degree of 20% with respect to the crosslinking. The density of the system is 𝜌 = 1.066 g/cm^3^. The normalized heat flux autocorrelation function (NHFACF) as function of the correlation time is illustrated in Figure 3b. As can be observed, a correlation length of 2.0 ps is adequate to obtain a decline to zero; thus, an equilibrated structure was reached in the time interval. Figure 3c illustrates the evolvement of the heat conductivity with correlation time. The converged final value of thermal conductivity for this uniform model structure can be derived as *λ* = 0.182 W/(m∙K). The value has been calculated at normal conditions for the temperature of 293.15 K and pressure of 1 atm. The MD run was performed for 500 ps with time steps of 0.2 fs.

A similar approach was applied to a random distribution of sulfur crosslinking in polybutadiene. The resulting model structure by combining head, repeat and tail groups, respectively, is shown in Figure 4a. The degree of crosslinking was again 20%, resulting in a corresponding density. For this kind of model system, a correlation length of 1.5 ps was adequate for the heat flux autocorrelation functions to obtain a decline to zero, as presented in Figure 4b. Thus again, the structures reached an equilibrium state that can be analyzed further. Figure 4c depicts the final converged value of the thermal conductivity for this model structure, and the value is calculated to *λ* = 0.242 W/(m∙K). The thermal conductivity has been calculated at normal conditions of temperature 293.15 K and pressure of *p* = 1 atm for 750 ps and time steps of 0.3 fs.

The comparison of the calculated final values of *λ* shows that there exists a significant impact of the crosslinking structure on the final heat conductivity of the model structure. Both having a similar density as well as overall sulfur content, the heat conductivity differs significantly beyond the simulation error. The obtained thermal conductivity for a polymeric structure with a uniform distribution of sulfur has a lower thermal conductivity than a polymeric structure with a random distribution of sulfur in the model structure. Therefore, the kind of distribution of crosslinkers in a polymeric model structure significantly influences the final value for thermal conductivities. 

In a subsequent phase, another polymer model structure with a lower degree of sulfur crosslinking was investigated to emphasize the influence of the number of sulfur bridges on the thermal conductivity of a final model polymer.

**Figure 5:**
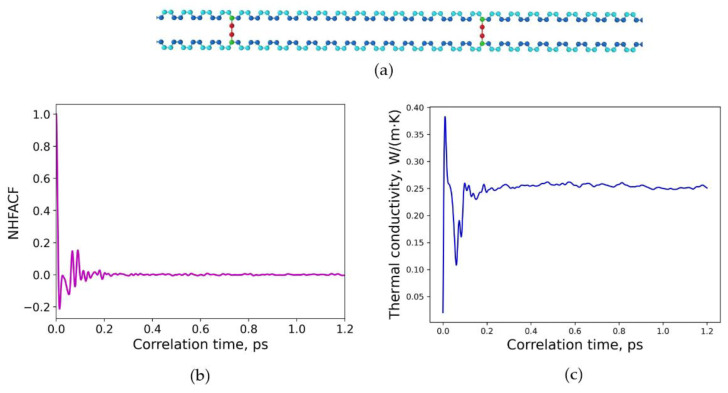
Random distribution of sulfur crosslinking in polybutadiene with longer distance between sulfur bridges: (**a**) molecular structure of one crosslinked chain, degree of crosslinking is 10.4%; (**b**) normalized heat flux autocorrelation function (NHFACF) as a function of correlation time with a correlation length of 1.2 ps; (**c**) thermal conductivity as a function of correlation time.

In the first attempt, a random distribution of sulfur crosslinking in polybutadiene with large distances between the sulfur bridges was considered, as shown in Figure 5a. The crosslinking degree of this structure was chosen to be 10.4%. The final density of this polymer model structure was 𝜌 = 1.019 g/cm^3^. As shown in Figure 5b, a correlation length of 1.2 ps was adequate to achieve a decline to zero for the heat flux autocorrelation functions. The converged value of the thermal conductivity for this model structure was calculated to *λ* = 0.252 W/(m∙K) and is given in Figure 5c. The thermal conductivity was calculated in the MD run at normal conditions for temperature 293.15 K and pressure of 1 atm for 600 ps and time steps of 0.3 fs.

**Figure 6:**
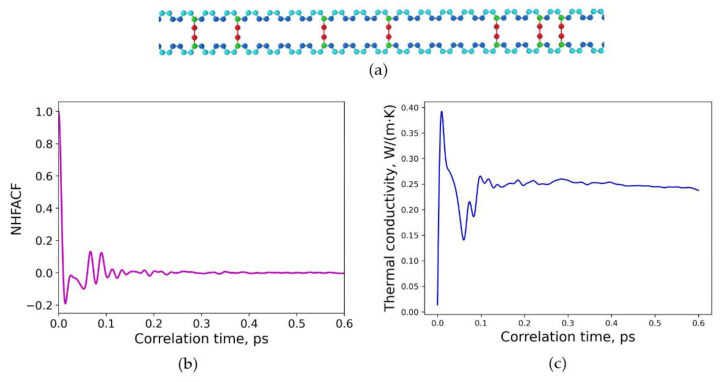
Random distribution of sulfur crosslinking in polybutadiene: (**a**) molecular structure of one crosslinked chain, degree of crosslinking is 30.4%; (**b**) normalized heat flux autocorrelation function (NHFACF) as a function of correlation time with a correlation length of 0.6 ps; (**c**) thermal conductivity as a function of correlation time.

Finally, two model structures with a very high degree of crosslinking were exemplarily investigated. First, in Figure 6a, a random distribution of the sulfur crosslinking in polybutadiene with a degree of crosslinking of 30.4% was considered. The distance between the sulfur bridges in this polymer model chain is smaller than the previous random model systems due to the higher sulfur content. The density of the polymeric model structure is 𝜌 = 1.144 g/cm^3^.

As can be seen in Figure 6b, the heat flux autocorrelation functions need a correlation length of 0.6 ps to achieve a final drop to zero. The converged value of the thermal conductivity for this model structure is calculated as *λ* = 0.234 W/(m∙K). The progression of the values with time is displayed in Figure 6c. The thermal conductivity has been obtained at normal conditions for a temperature of 293.15 K, a pressure of 1 atm for 900 ps and time steps of 0.2 fs.

**Figure 7:**
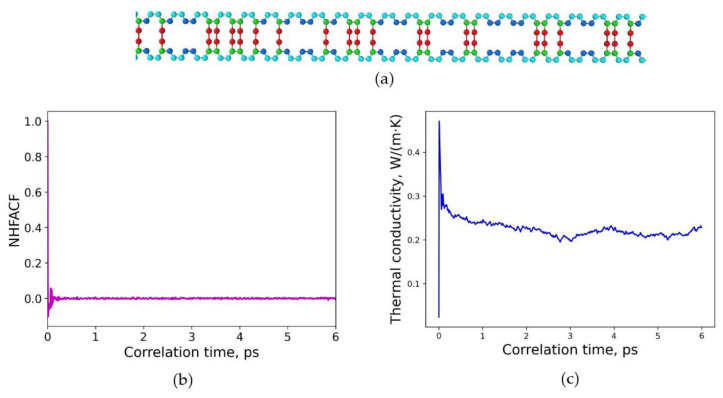
Random distribution of sulfur crosslinking in polybutadiene with extremely close distance between sulfur bridges: (**a**) molecular structure of one crosslinked chain, degree of crosslinking is 70%; (**b**) normalized heat flux autocorrelation function (NHFACF) as a function of correlation time with the correlation length of 6.0 ps; (**c**) thermal conductivity as a function of correlation time.

Increasing this high amount of sulfur further resulted in a model structure with a degree of crosslinking of 70%. In Figure 7, the random distribution of sulfur is illustrated. It is clearly visible that the amount of sulfur that is now given due to the combination of the head, repeat, and tail groups is higher compared to the other structures. The distance between sulfur bridges in these chains is extremely short in comparison to the polymer model chains in Figure 5 and Figure 6. A density of 𝜌 = 1.388 g/cm^3^ was calculated for this polymer model structure. In Figure 7b, a correlation length of 6.0 ps was found to be adequate to obtain a decline to zero for the heat flux autocorrelation functions. The converged value of the thermal conductivity for this model structure is *λ* = 0.218 W/(m∙K) and is shown in detail in Figure 7c. The thermal conductivity was calculated at normal conditions for a temperature of 293.15 K, a pressure of 1 atm for 600 ps and time steps of 0.3 fs.

Furthermore, for all studied structures above, the heat flux autocorrelation functions and thermal conductivities were studied along each space direction, e.g., *x*, *y* and *z*. By comparing them in each direction, it was observed that they have similar trends in all directions. Therefore, it can finally be concluded that all uniform and random model structures in the MD simulations were isotropic structures.

Various polymer–polybutadienes model systems addressing different kinds and degrees of sulfur crosslinking were investigated for comparison.

At first, uniform and non-uniform, e.g., random, model structures were evaluated. It was found that uniform model structures possess a very low value of heat conductivity compared to random crosslinked structures. As it may be more realistic to result from experimental vulcanizations in a random sulfur distribution, the degree of crosslinking was varied for this kind of crosslinking instead of using uniform models. Moreover, comparing the very low value of 0.18 W/(m∙K) of the uniform crosslinked model structure to those of random structures, the uniform crosslinked model seems to be more similar to un-crosslinked polybutadiene. It is worth noting that the thermal conductivity of pure polybutadiene through both doing EMD simulation and the transient measurements method was found to be *λ* = 0.18 W/(m∙K) and *λ* = 0.17 W/(m∙K), respectively, at normal conditions for the temperature of 293.15 K and a pressure of 1 atm [33]. Therefore, according to our obtained results, by adding sulfur to the polybutadiene base material at any degree of crosslinking, the final value of thermal conductivities increases significantly but only for random crosslinked structures, which may emphasize again that a uniform model structure may not be appropriate to describe crosslinked polybutadiene, as it may be closer to a crystallized fixed structure than model structures that have obviously more flexible parts in the polymer chains. Increasing the degree of crosslinking removes these flexible parts, thus resulting in reduced heat conductivity. 

The increase in heat conductivities from un-crosslinked to crosslinked polybutadiene structures is discussed in the literature and may be attributed to created covalent bonds during vulcanization, which produces a network of additional covalent bonds [38,39]; however, Rashidi et al. [40], by applying an MD simulation, showed that an enhancement in the value of the thermal conductivity by increasing the crosslinking degree is not specifically related to covalent bonds because the contribution of covalent bonds onto the value of the thermal conductivity was estimated to be only 20% for any density of crosslinking. Moreover, the crosslinking process will reduce the distance between the polymer chains by closing them to each other; therefore, by increasing the density of crosslinking the non-bonding coupling (vdW, Coulomb, or H-bonds) becomes more intensive, which in turn results in a thermal conductivity enhancement. 

**Figure 8:**
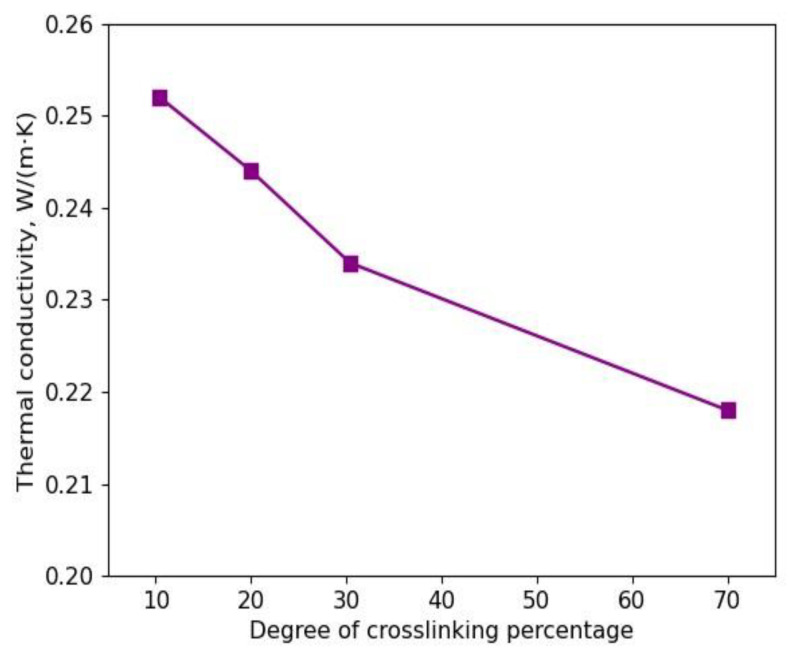
Converged thermal conductivity of randomly distributed sulfur crosslinked with polybutadiene as a function of percentage of crosslinking degree.

Thus, it is obvious that by adding a crosslinker to a pure polymeric structure, the thermal conductivity of the system would be increased; however, it does not necessarily mean that by increasing the degree of crosslinking, the thermal conductivity of the crosslinked polymeric structure will be increased as discussed in Ref. [41], because this correlation between the degree of crosslinking and the thermal conductivity depends on many more factors. One important factor would be that by increasing the degree of crosslinking, the degree of crystallization and periodicity of chains increases, which in turn results in the reduction in the thermal conductivity. This effect can be clearly seen when the degree of crosslinking for a random model structure changes. According to our results, four different random polymeric model structures with different degrees of crosslinking of 10.4%, 20%, 30.4%, and 70% (Figure 4, Figure 5, Figure 6 and Figure 7) were investigated in detail by MD simulations. The final converged thermal conductivities related to each degree of crosslinking are as follows: 0.252 W/(m∙K), 0.244 W/(m∙K), 0.234 W/(m∙K), and 0.218 W/(m∙K), as shown in Figure 8. Therefore, by increasing the degree of crosslinking from 10% to 70%, the thermal conductivity decreased significantly. Furthermore, crosslinking can counterintuitively reduce the amount of thermal conductivity. Yu et al. [42] experimentally surveyed that the thermal conductivity of HDPE was reduced by 30% because crosslinks negatively influenced the crystallization of the polymeric structure. Even though the enhancement of crosslinks can increase the inter-chain coupling intensity, the periodicity along the polymer chains can be broken by these crosslinks, which in turn results in intensive phonon scattering. Thus, it is obvious that by increasing the degree of sulfur crosslinking from 10% to 70%, the values of the thermal conductivities decrease. The influence of crosslinkers on the tuning of thermal conductivity is still an open question for scientists. 

Moreover, the mass percentages of polybutadiene in all the above polymeric structures were similar; however, the mass percentages of sulfur were different according to the increasing degree of crosslinking. The final densities of all above crosslinked polymeric model structures with different degrees of crosslinking—10.4% (Figure 3), 20% (Figure 5), 30.4% (Figure 6) and 70% (Figure 7)—were, respectively, as follows; 1.019 g/cm^3^, 1.066 g/cm^3^, 1.144 g/cm^3^ and 1.388 g/cm^3^. While applying the *NPT* ensemble, we continued *NPT* cycles to a point where the density becomes equilibrated. In the following *NVT* simulation, the mass of the polymeric structure and volume of the simulation box stayed constant. The corresponding densities of the simulated polymers were obtained by $\rho =m_{\mathrm{polymer}}/V_{\mathrm{box}}$. It can be observed that by increasing the degree of crosslinking of the polymeric structure, the density will be increased. Therefore, the density parameter may be considered as one additional factor to evaluate the percentage of the crosslinking degree in the structure, in regard to experimental studies aside from the heat conductivity.

## 4. Conclusions

The influence of the type and the degree of crosslinking on the thermal conductivity of polybutadiene crosslinked with sulfur as a function of the heat flux autocorrelation function was investigated in detail. The equilibrium molecular dynamic (EMD) simulation and Green–Kubo method were applied to calculate thermal conductivities for several model structures. In the first step, the influence of the type of crosslinking (uniform and non-uniform distribution of sulfur) on the thermal conductivity was studied. It was found that uniform model structures possess a very low value of heat conductivity compared to random crosslinked structures. The random distribution of crosslinker may be seen as a more realistic polymeric model with respect to real systems. In the second step, various polymeric structures with a random distribution of sulfur for different degrees of crosslinking 10.4%, 20%, 30.4%, and 70% were studied. By increasing the degree of crosslinking, the final values of thermal conductivities were found to be decreasing. One reason for that behavior may be that the periodicity along the chains would be broken, which in turn results in a reduced thermal conductivity. Moreover, the thermal conductivity of polybutadiene crosslinked with sulfur is much higher than that of pure polybutadiene, due to the creation of covalent bonds and increasing the inter-chain coupling. Moreover, by increasing the degree of crosslinking, the density of the polymeric structure is increased. Therefore, by measuring the density of the polymeric structure, the approximate degree of crosslinking can be obtained, which can be an efficient approach for experiments.

## Figures and Tables

**Table 1:** Force field parameters used in MD simulations. The usual factor of ½ in stretching and bending interactions is included in *K*_a_ and *K*_b_.

Force Field Parameters for Polybutadiene Crosslinked with Sulfur			
$U_{\mathrm{LJ}}=4\;\epsilon \;\left[{\left(\frac{\sigma }{r}\right)}^{12}-{\left(\frac{\sigma }{r}\right)}^{6}\right]$	$\epsilon \;\left[\frac{\mathrm{kcal}}{\mathrm{mol}}\right]$	σ [Å]	
$\left({\mathrm{CH}}_{3}-\right),\;\left(-{\;\mathrm{CH}}_{2}-\right)$	0.144	3.905	
$\left({\mathrm{CH}}_{3}-\right),\;\left(-\mathrm{CH}=\right)$	0.142	3.852	
$\left({\mathrm{CH}}_{3}-\right),\;(-\mathrm{CH}<)$	0.107	3.697	
$\left({\mathrm{CH}}_{3}-\right),\;\left(-S-S-\right)$	0.209	3.723	
$\left(-{\;\mathrm{CH}}_{2}-\right),\;\left(-\mathrm{CH}=\right)$	0.116	3.852	
$\left(-{\;\mathrm{CH}}_{2}-\right),\;(-\mathrm{CH}<)$	0.088	3.697	
$\left(-{\;\mathrm{CH}}_{2}-\right),\;\left(-S-S-\right)$	0.172	3.723	
(−CH=), (−CH<)	0.275	3.647	
(−CH=), (−S−S−)	0.170	3.673	
(−CH<), (−S−S−)	0.128	3.525	
$\left({\mathrm{CH}}_{3}-\right),\;\left({\mathrm{CH}}_{3}-\right)$	0.175	3.905	
$\left(-{\;\mathrm{CH}}_{2}-\right),\;\left(-{\;\mathrm{CH}}_{2}-\right)$	0.118	3.905	
(−CH=), (−CH=)	0.115	3.800	
(−CH<), (−CH<)	0.066	3.500	
(−S−S−), (−S−S−)	0.250	3.550	
$U_{\mathrm{bond}}=k_{b}\;{\left(r-r_{0}\right)}^{2}$	$k_{b}\left[\frac{\mathrm{kcal}}{\mathrm{mol}\;{Å}^{2}}\right]$	$r_{0}\;\left[Å\right]$	
$-{\mathrm{CH}}_{2}-{\mathrm{CH}}_{2}-$	260.0	1.526	
${\mathrm{CH}}_{3}-{\;\mathrm{CH}}_{2}-$	260.0	1.526	
$-{\mathrm{CH}}_{2}-\mathrm{CH}<$	260.0	1.526	
−S−S−	166.0	2.038	
−S−CH<	222.0	1.810	
−CH=CH−	530.0	1.340	
=CH−CH<	317.0	1.500	
$-{\;\mathrm{CH}}_{2}-\mathrm{CH}=$	317.0	1.500	
$U_{\mathrm{angle}}=k_{a}\;{\left(\theta -\theta_{0}\right)}^{2}$	$k_{a}\left[\frac{\mathrm{kcal}}{{\mathrm{mol}\;\mathrm{rad}}^{2}}\right]$	$\theta_{0}\;\left[\mathrm{degrees}\right]$	
−S−S−CH<	68.0	103.7	
$-\mathrm{CH}=\mathrm{CH}-{\mathrm{CH}}_{2}-$	70.0	118.0	
− CH=CH−CH<	70.0	118.0	
${\mathrm{CH}}_{3}-{\mathrm{CH}}_{2}-{\;\mathrm{CH}}_{2}-$	63.0	112.4	
$-{\mathrm{CH}}_{2}-{\mathrm{CH}}_{2}-\mathrm{CH}=$	---	---	
$=\mathrm{CH}-\mathrm{CH}\left(-S\right)-{\mathrm{CH}}_{2}-$	---	---	
=CH−CH(−S)−CH(−S)−	---	---	
=CH−CH(−S)−S−	---	---	
$U_{\mathrm{diherdal}}=\sum_{j=1}^{3}\left(\frac{K_{j}}{2}\;\left[1+{\left(-1\right)}^{j+1}\;\cos\left(j\varphi \right)\right]\right)$	$K_{1}\;\left[\frac{\mathrm{kcal}}{\mathrm{mol}}\right]$	$K_{2}\;\left[\frac{\mathrm{kcal}}{\mathrm{mol}}\right]$	$K_{3}\;\left[\frac{\mathrm{kcal}}{\mathrm{mol}}\right]$
${\mathrm{CH}}_{3}-{\;\mathrm{CH}}_{2}-{\mathrm{CH}}_{2}-\mathrm{CH}=$	–2.5	1.25	3.1
>CH−S−S−CH<	0.0	–7.414	1.705

## Data Availability

Not applicable.
